# Gestational diabetes: changed health beliefs in migrant women from five Asian countries living in Sweden: a prospective qualitative study

**DOI:** 10.1017/S1463423621000785

**Published:** 2022-01-12

**Authors:** Katarina Hjelm, Karin Bard, Jan Apelqvist

**Affiliations:** 1 Department of Public Health and Caring Sciences, Uppsala University, Uppsala, Sweden; 2 Department of Endocrinology, Division of Diabetes Care, Malmö University Hospital, Lund University, Malmö, Sweden

**Keywords:** beliefs about health/illness/health care, gestational diabetes, migrants/Asia, prospective study, qualitative study, semi-structured interviews

## Abstract

**Aim:**

The aim of this study was to explore the temporal development of beliefs about health, illness and health care in migrant women with gestational diabetes (GD) born in Asia residing in Sweden, and the influence on health-related behaviour in terms of self-care and seeking care.

**Background:**

Migrant Asian women are a high-risk group for developing GD. Adapting to the culture in the new society and the healthcare system, being diagnosed with GD and becoming a mother is demanding. The question is whether Asian migrants’ patterns of beliefs and behaviour change over time, as no previous study has been revealed on this topic.

**Method:**

Qualitative prospective exploratory study. Semi-structured interviews were held on three occasions: during pregnancy and three and fourteen months after delivery, with women born in Asia, diagnosed with GD. Data were analysed with qualitative content analysis.

**Findings:**

There was a temporal change of beliefs influencing health-related behaviour, showing a rising curve in risk awareness. An increasing number of persons described developing a healthy diet/lifestyle based on initial advice and shifted focus from the child to worries about the woman’s health and risk of developing type 2 diabetes and being unable to care for the child/family. Also, the number of women perceiving GD as a transient condition decreased and more believed it would last forever. Beliefs about health care were unchanged, the healthcare model was perceived working well but information about GD and follow-ups was requested even after delivery, and competent staff was expected. Health professionals’ beliefs about the seriousness of GD influence patients’ beliefs and need to be considered. Migrant women need support with adequate information, based on their individual beliefs, to continue develop a sustainable healthy lifestyle even after giving birth, to promote health and prevent type 2 diabetes.

## Introduction

Gestational diabetes (GD) implies health risks both for mother and child (Bellamy *et al*., 2009; Damm *et al*., 2016), and particularly for migrant women (Gagnon *et al*., 2011; Kragelund Nielsen *et al*., 2020) of Asian descent (Gagnon *et al*., 2011; Hedderson *et al*., 2012; Carolan, 2013a; Kragelund Nielsen *et al*., 2020). Migration to developed countries makes them a high-risk group due to ethnic origin (genetic susceptibility) and because they may adopt a more sedentary lifestyle and a calorie-dense low-fibre diet (environmental factors) leading to increasing rates of obesity. Socioeconomic factors (poverty), length of residency, pre- and postmigration experiences, length of residency, language and cultural beliefs also exert an influence. However, the risks of adverse outcomes can be improved by adopting preventive health behaviours (Veeraswamy *et al.*, 2012; Damm *et al*., 2016) which are based on individual beliefs about health and illness (Hjelm *et al.*, 2018). In contact with the new society and the healthcare system, the migrant will be confronted with the culture of the host country and the culture of the country of origin, and the encounter poses demands on adaptation (Berry, 2017), further increased by being diagnosed with GD and becoming a mother. Beliefs about health and illness, determining health-related behaviour, might then change. Only two former prospective investigations have been revealed studying the temporal development of beliefs in the new country (Hjelm *et al*., 2012; 2018), exploring women born in the Middle East and Africa, but none have been found focusing on migrant Asian women.

Pregnancy is a period of transformation and transition to motherhood, when a woman is responding to changes in body perceptions and adapting to mentally, physically and socially changes, which are further emphasised in women with GD (Craig *et al.*, 2020). GD is often connected to changes of lifestyle and emotional reactions due to the management and the perception of the condition being potentially life-threatening and as a disruption of pregnancy, negatively influencing quality of life (Parsons *et al.*, 2018). Yet despite a distressing (Jirojwong *et al.*, 2017; Parsons *et al.*, 2018; Craig *et al*., 2020) and shocking reaction to the diagnosis, a process of stepwise adaptation to the condition, facilitated by feelings of responsibility for the foetus, which gives motivation to follow advice from health professionals to manage themselves properly, is also described (Carolan, 2013b). Additional demands are made of migrant women as they have to adapt to life in a new country and culture in an acculturation process (Berry, 2017).

Previously migrant women with GD (Hjelm *et al*., 2012; 2018) and migrants diagnosed with diabetes mellitus of different origin (Dechamp-Le-Roux *et al*., 1990; Kulwicki, 1996; Hjelm *et al*., 2003) have described a more fatalistic view of causes of diabetes and an external locus of control, discussing the influence of fate or supernatural factors, and indicated limited knowledge and risk awareness of the disease. Higher risk of inadequate self-care because of limited health literacy and poorer understanding of gestational diabetes was shown in women of non-Caucasian origin in a multi-ethnic sample in a previous survey of attitudes to GD (Carolan *et al.*, 2010).

In Sweden, the migrant population is a mix of over 200 different nationalities, with Asians as the fourth largest non-European migrant group mainly including refugees from Vietnam, Afghanistan and Pakistan, and women immigrating from Thailand and China due to family ties (80% married to Swedish men) (Statistiska Centralbyrån (SCB), 2016).

## Aim

The aim of this study was to explore the temporal development of beliefs about health, illness and health care in migrant women with GD born in Asia residing in Sweden, and the influence on health-related behaviour in terms of self-care and seeking care.

### Health care for women with GD in Sweden

In Sweden, there are no national guidelines for screening and management of GD. Women in this study were screened for GD by a midwife at a healthcare centre in the 28th or 12th gestational week if they had heredity of diabetes mellitus or previously diagnosed GD. In case of testing positive, a referral was sent of the woman to the diabetes care team at the out-patient specialist diabetes clinic (Table 1). A diabetes specialist nurse provided basic information about management of GD and regular self-monitoring of blood glucose. Treatment was based on diet, reduced in sugar and fat and rich in fibres. Insulin was added if needed, and there were regular visits to the clinic. Information about GD was given 1–2 weeks later by a diabetologist, whereafter regular personal contacts with a diabetologist or diabetes specialist nurse (DSN) took place every third week during pregnancy in uncomplicated cases or individually prescribed. Those deemed in need were offered contact with a dietician for advice. In the case of dietary management, antenatal care was given at a healthcare centre by a midwife or at the maternity clinic if treated with insulin. Eight weeks postpartum, a midwife and/or an obstetrician was visited for family planning and a diabetologist was contacted if continued testing of blood glucose was needed. The last planned visit was about a year postpartum to a DSN for oral glucose tolerance test and lifestyle advice to prevent obesity.

**Table 1:** Health care for women diagnosed with gestational diabetes

Variable	During pregnancy	Postpartum	
		8 weeks	About a year
Healthcare staff and content	Team in diabetes care	Diabetologist	Diabetes specialist nurse
	Diabetologist (physician) responsible for the woman’s care	Check of health status and SMBG values if continued testing after delivery	OGTT
	Diabetes specialist nurse with an educating role initially		Lifestyle advice; importance of exercise and weight reduction by diet reduced in sugar and fat, rich in fibres (whole grains)
	Referral to dietician if deemed necessary		
Antenatal care	Midwife in a primary healthcare centre if treated with diet	Midwife and/or obstetrician for family planning advice	
	Midwife at the specialist maternity outpatient clinic if insulin-treated		
Postnatal care		Regular visits to the child healthcare clinic according to national programme for control of children’s health to paediatric specialist nurses and paediatricians	Regular visits to the child health care clinic according to national programme for control of children’s health to paediatric specialist nurses and paediatricians
Diabetes education	First visit to the clinic to a diabetes specialist nurse for additional investigations after initial screening. 1–2 weeks later information about GD from diabetologist Referral to dietician if deemed necessary Regular personal contacts with diabetologist and/or diabetes specialist nurse during pregnancy, or both Treatment was based on diet; reduced in sugar and fat, and rich in fibres (whole grains; keyhole marked products recommended). Insulin was added if needed, and the clinic was regularly visited.		
Regular check-ups	Personal contact with diabetologist or diabetes specialist nurse every third week in uncomplicated cases, or individually prescribed		
Self-monitoring of blood glucose (SMBG)	Four times daily every day if insulin-treated or every second day when treated with diet	Not on a routine basis	Not on a routine basis
Treatment with insulin	Individually prescribed		

OGTT = oral glucose tolerance test.

### Theoretical framework

Individual beliefs, for example about health and illness, are learned through socialisation by significant others in society and are culturally determined (Berger & Luckmann, 1991). Beliefs constitute a person’s attitudes guiding health-related behaviour and underpinned by knowledge (Glanz *et al.*, 2015). Beliefs about risks or risk awareness are additionally dependent on the trustworthiness of the information source (Glanz *et al.*, 2015). Different factors influence health-related behaviour; intra- and inter-personal, organisational or institutional, public and community policy, and a mutual relation between the individual and the environment is evident as he social environment both influences the behaviour and the reverse.

According to the theory of convergence (Gray *et al*., 2007), differences exist in health between generations as neither lifestyle nor behaviour is inherited. Thus, it is reasonable to presume changes over time in migrants as new demands are posed by the acculturation process on the individual attempting to adapt to the lifestyle and behaviour in the new society and the stress experienced may influence health beliefs, health behaviour and health. This is further influenced by being diagnosed with GD and the transition to motherhood. Health-related behaviour and degree of self-efficacy is affected by transitions, particularly those being stressful such as migration (Jerusalem & Mittag, 1995), experiences of mastery, mood (Bandura, 1995), cultural factors (Oettingen, 1995), perceived locus of control (Rotter, 1996), lay beliefs about explanations of disease (Helman, 2007) determining healthcare-seeking (Kleinman, 1980), perceived threat from a disease, including perceived susceptibility and severity of the condition (Rosenstock *et al.*, 1988), and sociodemographic factors.

## Methods

### Design

A qualitative prospective exploratory study with data collection by semi-structured interviews, on three different occasions, was completed. The interview style allowed participants to narrate their stories in order to get a deeper understanding from their own perspective (Flick, 2018).

### Participants

Included were nine Asian born women diagnosed with GD and residing in Sweden. Criterias for inclusion included age above 16 years and diagnosis of GD (O 24.4 – Gestational Diabetes Mellitus; in accordance with the IX version of International Classification of Diseases 1997). A consecutive sampling strategy was applied; staff at an in-hospital-based diabetes specialist clinic invited all female visitors with this diagnosis to participate in the study.

The informants were born in countries in Asia (Table 2), and their age range was 29–38 years (median 33 years). Four stated being refugees, and five had immigrated by family ties. Time of residence in Sweden was a median 8 years (range 1–17 years). With one exception, all women had been diagnosed with GD in Sweden and two had had GD previously. All, except three, women had been treated with diet only during pregnancy. Most were low educated (*n* = 6 < 9 years education), and some were dependent on social allowance and unemployed (*n* = 2 versus 3).

**Table 2:** Characteristics of the study population

Variable		
Country of origin (*n*)	*n*	Part of Asia^*^
Afghanistan	4	Southern Asia
Vietnam	2	South Eastern Asia
Thailand	1	South Eastern Asia
Pakistan	1	Southern Asia
China	1	Central or East Asia
Time of residence in Sweden (years)^†^	8 (1–17)	
Migrational background		
Refugee	4	
Family ties	5	
Educational level		
Primary school (<9 years)	6	
Secondary school (≥ 9 years)	3	

* According to UN world regions (2018).

† Values are median (range).

### Data collection

For each interview, a semi-structured thematic interview guide with open-ended questions was used. The first interview started with structured questions on background data. The interview guide was constructed based on a previous study (Hjelm *et al.*, 2012), review of literature and peer-review by diabetes specialist nurses, midwives and diabetologists experienced in management of women with GD.

Interviews were held in (1) gestational weeks 34–38; (2) three and; (3) fourteen months after delivery, and after pilot-testing of the interview guides in three women (excluded).

The interviews were held by a female diabetes specialist nurse (first author) not involved neither in the care of the women nor in the studied clinic. An authorised female interpreter was used if needed (in all except two cases speaking Swedish). According to the Swedish tradition (Hadziabdic & Hjelm, 2013), the sequential interpretation technique (interpreting word for word) was employed. The interviews were held outside the clinic in secluded rooms, continued about 1.5 h, were recorded and literally printed. The texts showed no non-translated sentences, were coherent, and thus showed good quality.

### Ethical considerations

The Ethics Committee of Lund University had approved the study (2010/280-31), written informed consent was obtained, and it was implemented in agreement with the Helsinki Declaration (World Medical Association, 2013).

### Data analysis

Data collection and analysis proceeded simultaneously until no new information appeared in the analysis (Flick, 2018). Content analysis was performed (Krippendorff, 2013), and by comparing the respondents’ opinions in each of the interviews, while also comparing the content between the three different interviews to study the temporal development, the search was for patterns and contradictions. The text was read through as a whole to get an overview and contextual information. Topics were identified by scrutinising the text line by line, where after the text was condensed into categories of content (Krippendorff, 2013). Thus, categories were developed inductively and titled as near the original text as possible. Categories from the theoretical models (Flick, 2018), the lay theory of illness causation (Helman, 2007) and the model of healthcare-seeking behaviour (Kleinman, 1980), were also introduced to the text. After each interview, the recordings were listened through and notes were made on the findings in general, new ideas, and themes.

### Rigour

The validity of the data was increased by triangulation of researchers in the analysis of data, and a diabetes specialist nurse and a general nurse (first and second author) were included. The content of the categorised data was checked by the first author (Krippendorff, 2013; Flick, 2018) and showed high level of agreement.

### Findings

Below, the numbers refer to interview number 1 during pregnancy, number 2 three months after delivery and number 3 fourteen months after delivery.

### Beliefs about illness

When talking about the temporal development of feelings from information about the diagnosis of GD, feelings of worries, especially for the baby, dominated at the first interview during pregnancy (Table 3). After delivery, worries changed to focus on own health, weight gain and a relapse of the condition.

1: I was very worried, I felt sick… All I thought about was what could happen to the baby. (S8 (number of respondent))

2: Before I did not know anything about complications and then I learned that this disease could affect the child…my weight could also be affected…as the doctor said that …I have been more worried…this machine that you check blood glucose with, is it possible to buy? I am still worried and want to control it. As the doctor said that when I am pregnant the more dangerous is it when it comes to diabetes and I have gained in weight…so I am worried. (S6)

3: Now I think that if I get pregnant again there will only be problems. I was actually scared. If I have a child with the same problem. So I was scared when I was pregnant that it would affect him…I don’t plan to get pregnant again. (S1)

**Table 3:** Development of beliefs about illness in migrant women with gestational diabetes (GD) born in Asia and living in Sweden

Variable	Gestational week 34–38	3 months after delivery	14 months after delivery
Emotions related to the diagnosis of GD	Worries, especially for the baby^†^	Worries for baby’s health decreased, instead worries for own health, weight gain and relapse of GD^†^	Worries for baby’s health decreased, instead worries for own health, weight gain and relapse of GD^†^
Thoughts about duration of GD	Don’t know	Don’t know	Don’t know
	The staff have said that it will disappear after delivery^†^	Some perceived the disease to be everlasting^*^	Even more claimed the disease will last forever^*^ Some said GD had gone^*^
Consequences of GD for health	Worries about the baby, feelings of uncertainty^†^	Own health a problem; gaining weight, sleep disorders, delivery related complications^*^	Tiredness, isolated, disturbed meals due to caring for a baby^†^
Problems related to GD Worries related to GD	Somatic symptoms; pollakisuria, tachycardia, pain in the body^*^ Fear and worry about the diabetes and the health of the child^*^,^†^	Somatic symptoms disappeared Worries related to development of diabetes and related complications^*^	Increased somatic symptoms; pollakisuria, tachycardia, pain in the body^*^ Increased worries for getting diabetes and the woman’s own health, relapse of GD, and especially worried about being unable to bring up the children^*^^,^^†^
Beliefs about future health	Worries about future health of the child^*^	Worries about future health in mother and child^*^ Hope for good health in mother and child Only God knows^‡^	Even more worries now, particularly for the woman’s own health but less for health of the child^*^ Don’t know

Categories in the lay theory model of illness causation by Helman (2007):

* Individual factors.

† Social factors.

‡ Supernatural factors.

were used as main analytical categories in data analysis when appropriate.

Most respondents claimed initially that GD would disappear, but over time an increasing number told it would last forever. Less told being informed by healthcare staff that GD was a transient condition between the first and third interview.

1: I don’t know but the doctor said it will last until the child is born. (S5)

3: It might perhaps still be in my body. I think it will be my entire lifetime….if I eat extra it will come. (S1)

When discussing consequences of GD for health, it was revealed that during pregnancy mostly worries about the baby were mentioned. After delivery, the focus had shifted to themselves and varying health problems; gaining weight, sleep disturbances, delivery-related complications, and in the last interview some stated being tired, isolated and meals being disturbed, all related to caring for a small child.

1: I fight for my baby….I don’t care so much about myself…I mostly think about the risk for the baby. (S8)

2: …I have gained weight. (S5)

2: I slept badly. (S7)

3: I have isolated myself at home… I feel tired and ill…actually been ill as I have gained weight … sit too much… (S1)

3: not enough sleep and meals are not regular as you have to care for the baby. (S7)

Most respondents expressed worries related to diabetes mellitus and development of complications at the second interview. In the last interview, the focus was on fears of being unable to care for the child because of illness or the risk of dying. Worries about relapse in GD were also brought up.

2: Diabetes is dangerous for the heart and the eyes, I don’t know whether it damages the skeleton. I think about my feet (wound). (S2)

3: I am scared something will happen to me and what then will happen to my children? …(S2)

3: This thought that it (GD) will be back again. (S3)

The main problems related to GD described by the women were somatic, for example polakisuria, tachycardia, pain in the body during pregnancy, which then had a U-shaped development after delivery with an increase again a year after delivery. However, factors harmful to health were stable over time and mainly focused on individual factors, such as stress and worries, and social factors, such as missing the family.

1: I frequently have to visit the toilet and …dry in my …mouth more often, the heart beats faster…pain in the body. (S2)

3: …the weight and the sight…felt I had some pain…my sight has become poorer. (S3)

1: Stress due to all the mail and so many bills…turn life upside down…problems they just send papers. (S1)

1: Just the thought of one’s family…mum and dad…want to see them, I miss them very much. (S3)

At the first and the last interview, most expressed worries related to GD. Over time, worries for the health of the child diminished, and at the two latter interviews these focused more on heredity and own health and risk of developing diabetes, and finally, at the last interview, the concerns were focused on ability to bring up the child.

1: I fight for my baby’s sake, I try to lose weight. I don’t care so much about myself but mostly …about the risk for the baby that, it will not affect him or her. (S8)

3: …only worried that I will get diabetes…I do care about my health for the sake of my children so that I can bring them up. (R)

At the interviews after delivery perspectives on future health evoked, and with a dominance of feelings of worry. At the last interview, these feelings were particularly directed at the woman herself and her health instead of the baby.

2: I want to be careful and try to avoid sweets or eating too much and bad dietary habits. I will try to …exercise so…I don’t want to get diabetes back. (S3)

3: When I think about the future and if it is possible after all that I or my children will get diabetes, that risk is there…I try my best by adapting our diet and physical exercise to avoid it…I think a lot about them (the children) and their health. (S6)

### Beliefs about health

Health during pregnancy was described predominantly as well-being and the ability to be active (individual factors). Health appeared to be increasingly associated with social factors such as the importance of health to be able care for the family and the children over time (see Table 4). In the second interview, one person also spoke about the influence of help from God (supernatural factor).

1: Health means everything…I need to feel healthy…feel well… able to do everything. (S8)

2: Health is a gift from God and now there is no problem…I am calm. don’t need to think about it and feel well mentally. (S6)

3: the most important thing…if you feel well…have health…no problems…then I can…take care of my children, the family, myself. But if you don’t feel well you can’t do anything. (S6)

**Table 4:** Development of beliefs about health in migrant women with gestational diabetes mellitus (GD) born in Asia and living in Sweden

Variable	Gestational week 34–38	3 months after delivery	14 months after delivery
Beliefs about health	Mental well-being^*^	Health is important to be able to take care of family and child^*^^,^^†^	Health is very important to be able to take care of the child^*^^,^^†^
	Ability to be active^*^		
Factors of importance for health	Diet^*^ Exercise^*^	Diet^*^ Exercise^*^	Diet^*^ Exercise^*^
		Achieving mental well-being is important^*^	Achieving mental well-being of even greater importance^*^
Factors negatively impacting health	Stress and worries^†^ Longing for their family^†^	Stress and worries^†^ Longing for their family^†^	Stress and worries^†^ Longing for their family^†^
Maintenance of health and prevention of complications	Diet/exercise^*^ Follow doctor’s advice^†^	Avoidance of worries^†^ Important to celebrate feasts/traditions and get together with other people^†^	Follow doctor’s advice^†^
Use of home remedies/alternative medicine to improve health	Very limited use^§^	Very limited use^§^	Very limited use^§^
Celebration of feasts/traditions important for health	Almost the same number spoke of positive and negative influence on health^*^	More talked about the negative influence on health, too much/wrong food^*^	Even more talked about the negative influence on health, too much/wrong food^*^
Beliefs about appropriate food	Sugar-reduced, low-fat, high-fibre diet	Back to ordinary diet/food habits before pregnancy, no sugar-reduction, more sugar, less fibres	High-fibre diet, back to initial healthy food advice during pregnancy
Beliefs about appropriate exercise	Taking walks is good	No advice about exercising but using different forms of exercise	No advice about exercising but using different forms of exercise
Influence of economy on health	DM diet considered more expensive than ordinary food but diet and health take priority over economy	Of no importance or influence for less than before	Even fewer considered economy of importance or influence for health than before

Categories in the lay theory model of illness causation by Helman (2007):

‡Supernatural factors.

* Individual factors.

† Social factors.

§ Factors related to nature were used as main analytical categories in data analysis when appropriate.

At all three interviews, diet and exercise (individual factors) were discussed as factors of importance for health. Trying to feel mentally well became more important over time at interview three.

1: I have to eat as they told me and… I have to walk two hours a day. (S5)

3: If you don’t have any problems (mentally, socially) health will be much better…I know if I start to …exercise, find some activities outdoors. (S1)

As harmful to health, ‘nothing’ was reported by some informants at all interviews while others talked about ‘stress’ and ‘longing for the family’. For maintaining health diet and exercise became more important over time while following doctor’s advice was mentioned in the first interview (during pregnancy) and then on the third occasion. In the second interview, social factors such as the importance of celebrating feasts and meeting other people were discussed as important for health.

1: I follow the rules…given…eat regularly. (S6)

2: They are important. These religious days… we meet …compatriots…that is good. (S5)

3: As my doctor told me…I must check my weight…I try to follow and take it in…also food and exercise, when you also have time for yourself. (S3)

Use of home remedies or alternative medicine measures was reported to a limited extent. All except two of the women were Muslims who said that ‘religion is important for health’ and ‘I pray and that means a lot…I feel calm’. An increasing number of women reported over time celebrations of feasts and traditions negatively influencing health by eating too much or inappropriate food. Others claimed it to be important for health as meeting others made them happy and others as unimportant for health. With one exception, none expressed having had either contact or information about self-help groups concerning diabetes or families.

During pregnancy and a year after delivery (first and third interview), a diet reduced in sugar and fat and rich in fibre was considered healthy and appropriate, while more women told that they ate ordinary food three months after delivery (second interview), but this decreased at the last interview. Eating a fibre-rich diet was in general described, and few stated reducing sugar intake in the second interview. More reported eating healthier again at the last interview.

1: …don’t eat so much fat food…much vegetables…I don’t eat any sweets either, no chocolate…or such…I eat proper food …not careless with what I eat. Eat fruit and vegetables…no chocolate, chips. (S 9)

2: …during pregnancy…it was good …to avoid sweets, eat dark bread…but that was expensive…keyhole (products)…I have used before…today I take everything…honey, marmalade, cheese, butter on ordinary bread, home-made. (S8)

3: before I was pregnant…I ate everything…Coca-Cola or Fanta…sweets and cookies…I baked all the time…But now nothing, I avoid all these…rice with less fat…porridge…for breakfast…fruit. (S6)

When comparing the situation at present, three months after delivery, with the one during pregnancy the majority described a regression to less healthier habits in life-style being less restrictive with diet and meals in contrast to a year after delivery when most had returned to the recommended diet (reduced in sugar and fat, rich in fibres) and/or ate less.

2: Before…I had control over everything I ate and diet was important and I ate regularly, but after delivery I have started to eat more…I’m not careful about with eating regularly, now I eat as I want…they said I should avoid eating sweets, drinking soft drinks, not drink so much milk…but I drink little milk compared to what I used to…but when it comes to food I permit myself everything. (S6)

3: I have talked to a dietician…told me how to eat…tried to eat much fruit and vegetables and less sugar…I continue with diet…I eat more vegetables…I buy special sugar for diabetic persons…easy to eat and tasty. (S7)

Thus, the women said they ate less and changed from sugar (sweets) and fat food into eating more vegetables and cooked food.

Some respondents, although fewer over time, perceived that the recommended diet (rich in fibres, reduced in sugar and fat) was more expensive than ordinary food while a few discussed the negative influence of poor economy on health, but put health before economy. The costs for, example, bread rich in fibres, keyhole-marked products (healthier fat, reduced in fat and sugar, rich in fibres (whole grains)), for example butter and sausages, were said [and is higher] to be almost twice as high.

Exercise was reported to be more frequent and varied in its forms at the second interview, but the majority had not received any advice about exercise after delivery.

2: You have to walk or exercise… you should cycle, skip and run outdoors. (S2)

3: I haven’t had any advice. When I was at check-up during pregnancy…the doctor said it’s important not to gain weight. (S4)

As regards self-monitoring of blood glucose, most respondents know when to use this during pregnancy and the frequency varied (4 times/day to every other day) while respondents with few exceptions had not received any advice about controls after delivery:

1: It was four times a day. Before food. (S7)

1: Until delivery. Every other day. (S4)

3: None (test of blood sugar). When I was pregnant I was offered to borrow this machine for self-test but not now. (S7)

The inclination to follow advice about diet and exercise decreased at the second interview, three months after delivery, and subsequently, the explanation was ‘I haven’t had any advice’ (S4).

### Beliefs about health care

Access to diabetes care and health care in general was described as easy but booking a visit to a physician was perceived difficult by some (see Table 5).

1: It is difficult to book a time (at the physician) but if you have a set time it’s easy. (S7)

2: …it’s easy to get there but…not so easy to get an appointment with a doctor. (S7)

2: Very difficult for me…I have doctors but I can’t go there. If I call they say, we don’t have any time. Then I hang up. (S2)

**Table 5:** Development of beliefs about health care in migrant women with gestational diabetes mellitus (GD) born in Asia and living in Sweden, inductively analysed

Variable	Gestational week 34–38	3 months after delivery	14 months after delivery
Access to health care	In general good but for some low access, difficult to get into contact with health care and book visit to physician	In general good but for some low access, difficult to get into contact with health care and book visit to physician	In general good but for some low access, difficult to get into contact with health care and book visit to physician
	Long waiting times	Long waiting times	Long waiting times
	Satisfaction with interpreters, most spoke Swedish	Satisfaction with interpreters, most spoke Swedish	Satisfaction with interpreters, most spoke Swedish
Communication with health professionals	Experienced as easy and unproblematic	Experienced as easy and unproblematic	Experienced as easy and unproblematic
Expectations on health professionals	–	Humble and respectful treatment	Humble and respectful treatment
		Professional competence important	Importance of good communication and regular follow-ups
Advice from the clinic concerning diet	Important to follow advice	Less inclination to follow advice	Returned to recommended diet Some ate less than during pregnancy
Advice from the clinic concerning exercise	Exercising	No perception of having received advice about exercise	No perception of having received advice about exercise
Advice from the clinic concerning SMBG	Several times/day, Varying/individual	None	None
Beliefs about the present healthcare model	Well-functioning	Well-functioning Lack of regular controls and information about GD, desire for more	Well-functioning Lack of regular controls and information about GD, desire for more
		Need for more dietary advice	Need for more dietary advice
		More information about where treatment is located at the hospital	
Expectations on health professionals: the ideal nurse or doctor	Positive to patients. Nice and helpful, humbly giving treatment. Communicating and having good contact.	Positive to patients. Nice and helpful, humbly giving treatment. Taking their time to listen	Positive to patients. Nice and helpful, humbly giving treatment. Taking their time to listen
	Professional competence. Good ability to inform patients	Professional competence Good ability to inform patients	Professional competence Good ability to inform patients

Communication/contact with health professionals was described as easy and unproblematic. The need for an interpreter, either a professional or a relative (husband), varied, and all but two could manage in Swedish. One person stated in the last interview the need for an interpreter but had not had access to any.

After delivery, the inclination to follow advice changed over time.

1: I got advice to eat vegetables, liver and less sugar. (S7)

Interviewer: Did you follow the advice?

1: Yes. (S7)

3: During pregnancy I ate good food, I ate a little food many times a day. But nowadays I don’t eat so much vegetables…mostly meat…when the kids are at home I have to make pancakes or French toast. When eating in a restaurant…lots of hamburgers and Cola light. (S1)

3: Interviewer: Do you follow advice?

No. I don’t think I can get diabetes…I had it during pregnancy but it went away§. (S4)

The perception of health professionals being important for health as they ‘give information’ was stable over time while ‘offering help’ became more frequent and ‘don’t know’ less often stated three and fourteen months after delivery.

An ideal, or good, nurse or physician was described as a person with a positive attitude to the patient by being ‘nice, considerate, and having good contact’ and in the interviews after delivery ‘taking their time to listen’ was added. On all three occasions, professional competence in terms of a ‘good ability to inform’ patients was also discussed.

The present healthcare model was stated as working well at all interviews, and the women expressed getting help needed and thus did not lack anything. The expectations on health professionals at all interviews mainly concerned the attitude towards the patient being positive and the wish they should be ‘nice to me’, and at the final interview the focus on professional competence in terms of knowledge and regular check-ups of the disease was added.

The informants experienced, with few exceptions, no support in managing their health from health care after delivery (3 and 14 months) but desired information, regular control and help/support when ill.

2: Now I don’t get any help or support from health care. (S3)

3: …that they could keep us a week in hospital and teach us how to eat…or how blood glucose works. (S1)

3: …if I have a disease then I want help. (S7)

## Discussion

This investigation is unique as it explores the temporal development of beliefs about health, illness and health care in migrant Asian women with GD, from during pregnancy up to about a year after delivery. Comparisons to former studies are thus limited.

The main result was a change of beliefs over time, with increased risk awareness and women describing how they developed and practised a healthy diet/lifestyle based on initial advice and a shift in focus to worries about the woman’s own health and the risk of developing type 2 diabetes and then not being able to care for the child and the family. Also, those who perceived GD as passing decreased and more believed it would last forever. Beliefs about health care did not change over time and the healthcare model was found to function well, but information about GD and its management and regular follow-ups were requested.

### Beliefs about health and illness

The increased risk perception over time and the change in beliefs in migrant Asian women with GD from focusing on individual factors and well-being to emphasising social factors (Helman, 2007), such as being able to care for the child and the mother’s own health, are associated with various factors such as the process of becoming a mother and adapt to and understanding the meaning of it and also being responsible for a child (Craig *et al.*, 2020); thoughts about the baby’s health have been shown to be a strong motivator for adherence to management regimens for women with GD (Carolan, 2013b); children are of great importance for women; the test of oral glucose tolerance a year after birth reminds about the susceptibility to GD (Rosenstock *et al.*, 1988); and questions related to GD posed by the researcher (diabetes specialist nurse) in three encounters imply an unplanned intervention effect, impacting beliefs in both patients and healthcare staff (Glanz *et al*., 2015; Flick, 2018). There is a mutual relation between the individual and the environment, and the social environment both influences the behaviour and the reverse (Glanz *et al*., 2015).

The change in focus from the health of the baby to the health of the mother and her risk of developing type 2 diabetes might be related to becoming a mother, and GD has a significant impact on the woman’s life (Carolan, 2013b; Craig *et al*., 2020) as it is perceived as a distressing disruption of a normal pregnancy (Jirojwong *et al.*, 2017; Parsons *et al*., 2018; Craig *et al*., 2020) and being life-threatening (Parsons *et al.*, 2018). The birth of a healthy baby is then felt as a relief (Parsons *et al*., 2018) demonstrating the reverse. The medicalisation of pregnancies due to societies being more risk-focused (Parsons *et al.*, 2018) experienced seriousness of disease by healthcare staff and in the healthcare system (Dunning & Martin 1999; Hjelm *et al.*, 2008) are also influencing. Respondents in this study said they were informed by health professionals about the transience of GD, and the check-up six weeks postpartum did not focus on GD, nor was it delivered by a diabetes specialist nurse; instead, information about family planning was delivered by a midwife and an obstetrician having another focus.

The migrant Asian women did not express worries like those shown in migrant African women about the future risk of being bound to problematic changes of diet (Hjelm *et al.*, 2018), or being uncertain about what traditional food to eat, or having difficulties in abandon traditional habits (Wallin *et al*., 2007), or the stress particularly related to adjustment of dietary habits described in studies on women’s experiences of GD (Devsam *et al.*, 2013; Craig *et al.*, 2020) and dietary management (Leung Hui *et al.*, 2014; Craig *et al*., 2020). Irrespective of expressed lack of information about diet from health professionals postpartum, respondents described a positive change of beliefs about dietary adjustment to a healthier diet from eating more sweets and fat before pregnancy to eating more vegetables and cooked food a year after delivery, and thus, there is a need for support in developing a healthy lifestyle in women with GD (Van Ryswyk *et al*., 2015), with a particular focus on diet. Many of the respondents perceived the recommended diet to be expensive and thus need information on how to develop healthy dietary habits at low cost and alternatives to expensive keyhole products, particularly as most were low-educated, unemployed or dependent on social allowances. Healthcare staff need to consider the influence of socioeconomic factors in their advice, as poverty is an important environmental risk factor for health, particularly in migrants of Asian origin having a heightened genetic susceptibility (Carolan, 2013a; Kragelund Nielsen *et al.*, 2020). The risk of adapting a sedentary lifestyle and a calorie-dense low-fibre diet (environmental factors), leading to increasing rates of obesity, needs to be prevented by adequate information as early as possible.

The difference from the previous studies of Africans (Wallin *et al*., 2007; Hjelm *et al.*, 2018) might be related to dissimilarities in cultural distance, defined as differences in cultural values, living standards including food habits, social structure and religion (Triandis, 2000). The distance may possibly be shorter for Asians with a lower barrier to change of habits (Rosenstock *et al*., 1988). Another explanation is an increased number of women in this study reporting somatic symptoms a year after delivery, perceived as consequences of GD, which might remind them of the seriousness, perceived threat and vulnerability of the condition, thus making it visible and motivating active health behaviour (Rosenstock *et al*., 1988).

Health was expressed as feelings of well-being and not focused on freedom from disease as stated in migrant women with GD from the Middle East and Africa (Hjelm *et al*., 2012; 2018). Nor was there a confirmation of influence of supernatural factors (Allah, God) or fate leading to an external locus of control (Kulwicki, 1996; Hjelm *et al*., 2003). Instead, a change was shown towards an internal locus of control (Rotter, 1996), expressed in a wish for more information about handling GD and a continuous seeking of information as in women from the Middle East (Hjelm *et al.*, 2003; 2005).

Beliefs about health and illness in the migrant Asian women developed over time, demonstrating an increasing risk awareness and healthy diet/lifestyle habits in contrast to a U-shaped development in migrant Middle Eastern women, with a regression to poorer habits after giving birth (Hjelm *et al*., 2012), and an almost unchanged low-risk awareness and limited knowledge prospectively in migrant African women (Hjelm *et al.*, 2018). Thus, beliefs are associated with cultural and situational factors (Helman, 2007; Glanz *et al*., 2015), individual (Helman, 2007) and need to be appraised for planning of individualised care.

### Beliefs about health care

Access to diabetes care and health care in general was perceived as easy, except booking physician visits, contrary to a previous prospective study of migrant women with GD from the Middle East (Hjelm *et al.*, 2012). The difference might be due to the fact that most women in this study were Swedish-speaking or assisted by their husband and thus not in need of an interpreter as women from the Middle East. However, the healthcare model evaluated was experienced as working well, with the exception of lack of reported regular follow-ups and information about GD, but irrespective of this additional care was not searched for outside institutions in the professional sector (Kleinman, 1980). Many felt left with questions and wished, particularly in the postpartum period, for staff taking time to provide information about GD supporting previous findings of lack of appropriate information (Lindmark *et al*., 2010). Perceived lack of clear explanations has been reported to evoke emotions of hostility/anger (Glanz *et al*., 2015), negatively affecting, not only the blood glucose level but also the feeling of self-efficacy governing self-care activity (Bandura, 1995).

Expectations on healthcare staff were unchanged over time, and the significance of professional competence in communication and taking time to inform the patients also after giving birth was emphasised, as previously found (Hjelm *et al*., 2012; 2018). Then, it is important to inform patients that GD may be a transient condition but foremost that preventive measures are needed to maintain health in both mother and child (Veeraswamy *et al*., 2012; Damm *et al*., 2016). Expectant women, especially migrant women with GD in the new country, are vulnerable and need support in the process of becoming a mother, to concentrate on health of themselves and the child by adequate and repeated information during pregnancy, postpartum and onwards based on their individual beliefs. Implications of the study are summarised in Table 6.

**Table 6:** Summary of implications of the findings

• Beliefs about the seriousness of the disease in healthcare staff and the healthcare organisation influence patients’ beliefs.
• Healthcare staff with professional competence in communication, taking their time to inform the patients, and assess individual beliefs about health and illness are needed.
• Access to credible sources to get information about appropriate management of GD and the long-term implications on women’s health of having GD is important to prevent perceived lack of clear explanations leading to stress.
• It is important to inform patients that GD may be a transient condition and that preventive measures are needed to maintain health in both mother and child.
• Support in developing a healthy lifestyle in women with GD, with a particular focus on diet, is needed.
• Migrant mothers need support in the process of becoming a mother, to concentrate on health of themselves and the child by adequate and repeated information, based on their individual beliefs, during pregnancy, postpartum and onwards.

### Limitations

A consecutive sampling strategy had to be used as no registers on women diagnosed with GD born abroad existed. The sample included mainly low-educated women (<9 years) but also some with education from upper secondary school. Level of education might impact beliefs about health and health-related behaviour (Rosenstock *et al.*, 1988) but within-group analysis did not show any dissimilarities. Women from different countries in Asia, although mostly from the southern part, with different time of living in Sweden and migrational backgrounds were included. Those are components that can affect beliefs about health and care-seeking behaviour, and being unable to decide the causal factor could be seen as a limitation. The group investigated is to be seen as mirroring the population of migrant women from Asia who are to be found in a Swedish maternity clinic. A qualitative exploratory study design was used to find different perspectives and try to understand the informants’ views instead of explaining results being generalisable to a wider population (Flick, 2018). However, data showed a homogenous picture, and carefully collected, analysed and described, the findings can be transferred to similar persons or contexts (Krippendorff, 2013).

## Conclusions

There was a temporal change in beliefs about health and illness influencing health-related behaviour, showing a rising curve in risk awareness and development of a healthy diet/lifestyle. Beliefs about the seriousness of the disease in healthcare staff and the healthcare organisation influence patients’ beliefs. It is important to consider the influence of social environment on behaviour and support migrant women with adequate information, based on their individual beliefs, to continue develop a healthy lifestyle even after giving birth, in order to promote health and prevent type 2 diabetes.
